# Emerging molecular mechanisms in chemotherapy: Ca^2+^ signaling at the mitochondria-associated endoplasmic reticulum membranes

**DOI:** 10.1038/s41419-017-0179-0

**Published:** 2018-02-28

**Authors:** Martijn Kerkhofs, Mart Bittremieux, Giampaolo Morciano, Carlotta Giorgi, Paolo Pinton, Jan B. Parys, Geert Bultynck

**Affiliations:** 1 Department of Cellular and Molecular Medicine and Leuven Kanker Instituut, KU Leuven, Laboratory of Molecular and Cellular Signaling, Leuven, Belgium; 2 Department of Morphology, Surgery and Experimental Medicine, Section of Pathology, Oncology and Experimental Biology, Laboratory for Technologies of Advanced Therapies (LTTA), University of Ferrara, Ferrara, Italy; 3 Cecilia Hospital, GVM Care & Research, E.S: Health Science Foundation, Cotignola, Italy; 4 CNR Institute of Cell Biology and Neurobiology, Monterotondo, Italy

## Abstract

Inter-organellar communication often takes the form of Ca^2+^ signals. These Ca^2+^ signals originate from the endoplasmic reticulum (ER) and regulate different cellular processes like metabolism, fertilization, migration, and cell fate. A prime target for Ca^2+^ signals are the mitochondria. ER–mitochondrial Ca^2+^ transfer is possible through the existence of mitochondria-associated ER membranes (MAMs), ER structures that are in the proximity of the mitochondria. This creates a micro-domain in which the Ca^2+^ concentrations are manifold higher than in the cytosol, allowing for rapid mitochondrial Ca^2+^ uptake. In the mitochondria, the Ca^2+^ signal is decoded differentially depending on its spatiotemporal characteristics. While Ca^2+^ oscillations stimulate metabolism and constitute pro-survival signaling, mitochondrial Ca^2+^ overload results in apoptosis. Many chemotherapeutics depend on efficient ER–mitochondrial Ca^2+^ signaling to exert their function. However, several oncogenes and tumor suppressors present in the MAMs can alter Ca^2+^ signaling in cancer cells, rendering chemotherapeutics ineffective. In this review, we will discuss recent studies that connect ER–mitochondrial Ca^2+^ transfer, tumor suppressors and oncogenes at the MAMs, and chemotherapy.

## Facts

- Ca^2+^ fluxes between ER and mitochondria affect several cancer hallmarks, including apoptosis resistance, migration, and invasion.
- Oncogenes and tumor suppressors residing at the MAMs execute part of their cellular function by altering ER–mitochondrial Ca^2+^ transfer, thereby promoting or preventing cancer cell survival.
- Dependent on the cancer type and cancer stage, ER–mitochondrial Ca^2+^ transfer can either exert anti-tumorigenic effects like restoring apoptosis sensitivity or exert pro-tumorigenic effects like promoting metastatic behavior.
- Different chemotherapeutics rely on a Ca^2+^-signaling component to induce cancer cell death.
- Ca^2+^ signaling modulation can (re)sensitize or increase the responsiveness of cancer cells towards chemotherapeutics.

## Open questions

- How can Ca^2+^ signaling at ER–mitochondrial contact sites be modulated in a cancer-specific manner to fight cancer cell survival?
- Can ER–mitochondrial Ca^2+^ signaling events overcome dysregulated cell survival/apoptosis sensitivity in cells with altered oncogene and/or tumor suppressor function?
- What processes/regulation pathways underlie or control differences between ER–mitochondrial Ca^2+^ transfer in cancer cells vs. normal cells?
- How can Ca^2+^-signaling modulation be applied to increase responsiveness and sensitivity to existing therapies and to induce cancer cell-specific cell death while sparing normal cells?
- Can Ca^2+^ signaling be applied in a cancer stage-specific manner, thereby promoting cell death and avoiding metastasis?
- What other molecular mechanisms, like the generation of ROS, exchange of lipids or alterations in protein composition, or ER–mitochondrial tethering at the MAMs impact or cooperate with Ca^2+^ signaling in anti-cancer chemotherapeutic actions?

## Introduction: ER–mitochondrial Ca^2+^ signaling in cell death and survival

Mitochondria do not only fulfill the function of powerhouse of the cell but their function also encompasses more than merely providing the cell with ATP^1,2^. Currently, mitochondrial function has been implicated in apoptosis, autophagy, cell proliferation, cellular senescence, and migration^3–6^. Furthermore, mitochondrial function is impacted by the “state” of the mitochondrial network, which can range from highly connected to fragmented^7^. Nevertheless, mitochondria do not act as sole orchestrators of cellular processes. In fact, the mitochondrial network rather functions as a highly versatile signaling platform, closely connected to other cell organelles, like the endoplasmic reticulum (ER)^8^ and peroxisomes^9^. To allow for inter-organellar cross-talk, the different organelles are often located in close proximity to each other^10,11^, like the ER and the mitochondria, which are connected through mitochondria-associated ER membranes (MAMs). These MAMs are defined as ER membranes that are in close apposition (10–50 nm) to the mitochondria and were first isolated as a distinct entity in the early 1990s^11–13^. In recent years, MAMs were shown to contribute to various cellular functions like metabolism, autophagy, lipid synthesis but also cell survival and cell death^8,14–18^. In this sense, the MAMs, like mitochondria, are highly dynamic signaling hubs where signals from different cellular pathways converge and are integrated^15,19–21^.

One of the signals transferred between ER and mitochondria at the MAMs is the ubiquitous second messenger Ca^2+^^22,23^. While [Ca^2+^] in the cytosol is maintained at low levels under resting conditions, the bulk of intracellular Ca^2+^ is confined in the ER^22^. Ca^2+^ is predominantly released from the ER via the inositol 1,4,5-trisphosphate (IP_3_) receptor (IP_3_R), which is gated by IP_3_^24^, or the ryanodine receptor (RyR)^25^. However, Ca^2+^ accumulation into the mitochondrial matrix requires Ca^2+^ transport across the outer mitochondrial membrane (OMM) and the inner mitochondrial membrane (IMM). At the OMM, Ca^2+^ transport is mediated via the high-conductance voltage-dependent anion channel 1 (VDAC1), while at the IMM, Ca^2+^ transport is mediated via the mitochondrial Ca^2+^ uniporter (MCU), the pore-forming unit in the MCU complex, consisting of MCU itself and its regulators^26–28^. For a detailed description of MCU regulation we would like to refer to refs. ^29,30^. The MAMs play an important role in mitochondrial Ca^2+^ uptake, since they provide a Ca^2+^ micro-domain, where Ca^2+^ levels are higher than in the bulk cytosol^1,15^. This is necessary to sustain ER–mitochondrial Ca^2+^ signaling since the MCU has a low affinity for Ca^2+^. Thus, the MAMs allow for efficient, ‘quasi-synaptic’ mitochondrial Ca^2+^ uptake upon ER Ca^2+^ release through the formation of a micro-domain^1,15,31^. This emphasizes the importance of the MAMs as a signaling hotspot.

**Fig. 1:**
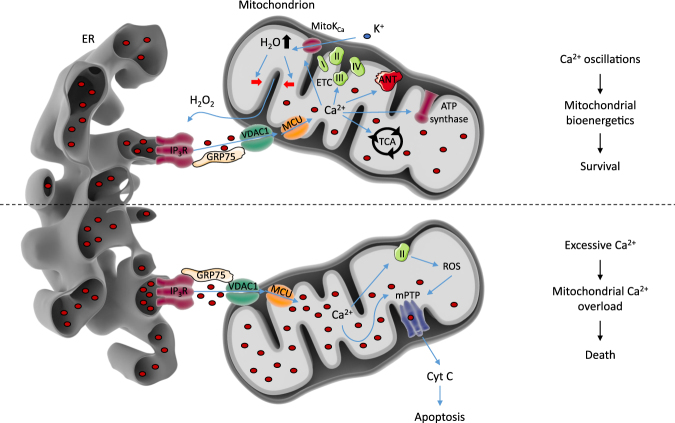
Ca^2+^ signaling at the ER and the mitochondria in cell death and survival. Arrow-headed lines indicate a stimulatory or consequential effect. The ER is the main intracellular Ca^2+^ storage organelle. The release of Ca^2+^ from this organelle is mediated by the IP_3_R, gated by the intracellular messenger IP_3_. Ca^2+^ then travels via VDAC1, which is physically coupled to the IP_3_R through GRP75, and MCU to the mitochondrial matrix. Ca^2+^ oscillations targeted to the mitochondria are able to stimulate mitochondrial metabolism in several ways. Firstly, the TCA cycle has three rate-limiting enzymes that are regulated by Ca^2+^: pyruvate dehydrogenase, isocitrate dehydrogenase, and α-ketoglutarate dehydrogenase. Furthermore, both the ATP synthase and complex III of the electron transport chain (ETC) are stimulated by Ca^2+^. In addition, the adenine nucleotide translocase (ANT) is activated as well. Interestingly, positive feedback mechanisms exist to ensure Ca^2+^ feeding into the mitochondria. One of these mechanisms is dependent on a redox-nano-domain at the MAMs: Ca^2+^ influx into the mitochondrial matrix activates Ca^2+^-activated K^+^ channels and parallel H_2_O uptake in the mitochondria. This results in cristae compression (indicated by the red arrows) and H_2_O_2_ extrusion, which stimulates IP_3_R activity. In short, by stimulating cellular metabolism, Ca^2+^ oscillations contribute to cell survival. However, excessive Ca^2+^ uptake in the mitochondria causes mitochondrial Ca^2+^ overload. This results in opening of the mPTP, either by a direct action of Ca^2+^ on the mPTP or by Ca^2+^ binding to cardiolipin, thereby disrupting complex II of the ETC and subsequent ROS production. mPTP opening leads to mitochondrial swelling, rupture of the OMM, and release of pro-apoptotic factors like cytochrome *c* and ultimately apoptosis

Mitochondrial Ca^2+^ signals are decoded differentially depending on their spatiotemporal characteristics (see Fig. 1). For example, cytosolic Ca^2+^ oscillations, efficiently transferred to the mitochondria through these contacts sites, drive mitochondrial metabolism. Moreover, several mechanisms account for the dynamic interplay between Ca^2+^ signals and mitochondrial metabolism. Ca^2+^ increases the activity of several rate-limiting enzymes of the tricarboxylic acid (TCA) cycle, including pyruvate, isocitrate, and α-ketoglutarate dehydrogenases^32^, while MCU transcription is controlled by the cAMP-responsive element binding protein, a Ca^2+^-dependent transcription factor^33^. Cells can also fine tune the level of Ca^2+^ oscillations that drive mitochondrial bioenergetics via a redox-nano-domain^34^. Mitochondrial Ca^2+^ uptake triggers K^+^- and H_2_O-influx into the mitochondrial matrix, leading to cristae compression and H_2_O_2_ release at the MAMs. This provides positive feedback on IP_3_Rs, enhancing their ability to sustain Ca^2+^ oscillations^34^. Other contributions of Ca^2+^ to cell metabolism are the stimulation of complex III of the electron transport chain, as well as stimulation of the ATP synthase and the adenine nucleotide translocase^35^.

Ca^2+^ oscillations in the cytosol can also modify mitochondrial metabolism indirectly by activating ARALAR, a mitochondrial glutamate/aspartate transporter playing a central role in the malate/aspartate shuttle, which is strongly dependent on cytosolic Ca^2+^ signaling^36^. Ca^2+^ binds to ARALAR and activates ARALAR-mediated glutamate and NAD(P)H transport into the mitochondria^36–38^. Also, pyruvate production is Ca^2+^ dependent and is linked to the malate/aspartate shuttle, providing pyruvate to the mitochondria^39,40^. In addition, there is tissue-specific regulation, which determines the threshold for mitochondrial Ca^2+^ influx and its effects^41^. Liver cells and cardiomyocytes contain a high and a low ratio of mitochondrial Ca^2+^ uptake 1 (MICU1)/MCU, respectively, with MICU1 (together with MICU2) being an MCU regulator which affects the cooperativity of mitochondrial Ca^2+^ uptake^42,43^. Therefore liver cells display a high cooperativity of mitochondrial Ca^2+^ uptake, while cardiac cells display a low cooperativity. This prevents the occurrence of mitochondrial Ca^2+^ signals in response to short-lasting Ca^2+^ transients and thus avoids mitochondrial Ca^2+^ overload even when the heart beats at high frequency^41^. Recently, it was shown that apart from affecting the cooperativity of mitochondrial Ca^2+^ uptake, MICU1, together with its paralog MICU2, inhibits the MCU at cytosolic Ca^2+^ concentrations lower than ~600 nm, thereby determining the relatively high threshold for MCU-mediated Ca^2+^ uptake^44^. Compared to wild-type cells, loss of MICU1, which also results in loss of MICU2, lowers the threshold for MCU-mediated mitochondrial Ca^2+^ uptake to about 200 nM Ca^2+^. Compared to mitochondria lacking both MICU1 and MICU2, mitochondria containing MICU1 but not MICU2 display a higher threshold for mitochondrial Ca^2+^ uptake (~350 nM Ca^2+^), indicating that MICU1 by itself can inhibit MCU. These observations indicate that MICU1 does not only control MCU cooperativity^43^, but can also function as a gatekeeper of MCU^44^. Moreover, MICU2 requires MICU1 to regulate MCU. MICU1’s function as a gatekeeper is also important in vivo to prevent mitochondrial Ca^2+^ overload, as evidenced in MICU1-knockout animals, developing ataxia and muscle fatigue associated with elevated mitochondrial Ca^2+^ levels and reduced ATP levels^45^. Finally, MICU1 and MICU2 were shown to bind the mitochondrial lipid cardiolipin, facilitating membrane anchoring of the complex and the fine-tuned Ca^2+^-dependent regulation of the MCU by MICU1 and associated factors, like EMRE^44^.

On the other hand, massive Ca^2+^ release from the ER causes mitochondrial Ca^2+^ overload, resulting in opening of the mitochondrial permeability transition pore (mPTP)^46,47^, mitochondrial swelling, and subsequent cell death (Fig. 1)^48–51^. Several mechanisms may account for this. For instance, mitochondrial Ca^2+^ influx results in Ca^2+^ binding to cardiolipin, causing the disintegration of complex II and the release of the functionally active catalytic subunits in the mitochondrial matrix, providing a source of reactive oxygen species (ROS) that triggers the opening of the mPTP^52^. In addition to this, recent evidence emerged that mitochondrial Ca^2+^ could directly target the mPTP, resulting in a conformational change leading to its opening and to subsequent mitochondrial swelling^53^.

## Regulation of ER–mitochondrial Ca^2+^ signaling at the MAMs

**Fig. 2:**
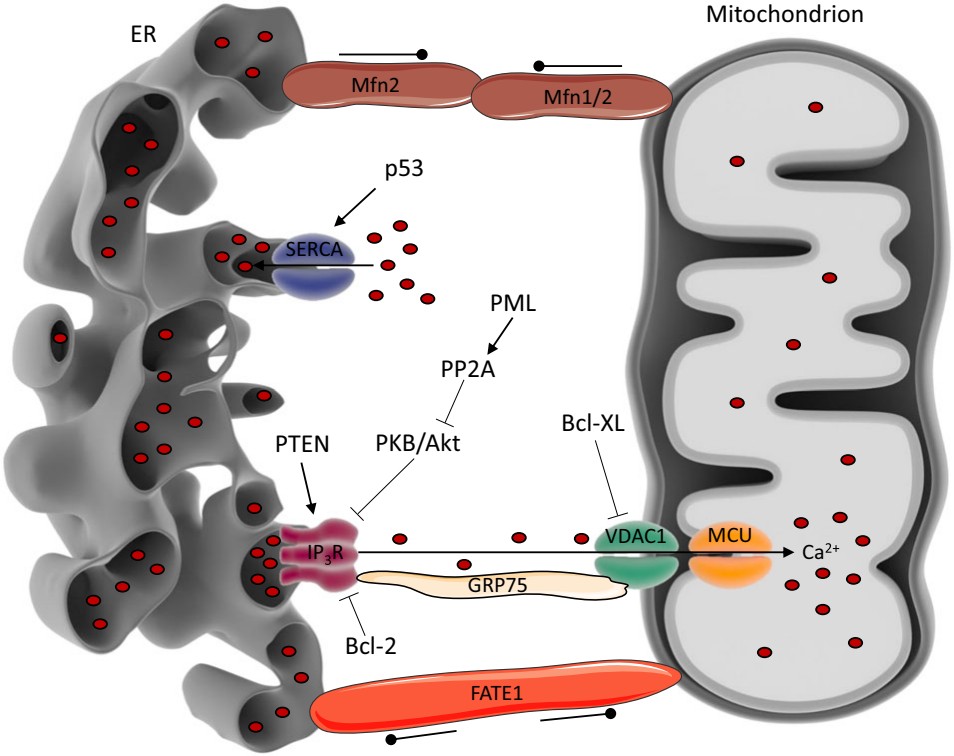
Regulation of ER–mitochondrial Ca^2+^ signaling by oncogenes and tumor suppressors at the MAMs. Arrow-headed full lines indicate a stimulatory effect, while bar-headed full lines indicate an inhibitory effect. Dot-headed lines indicate a tethering or a spacing effect, depending on the inward- or outward-facing dots, respectively. ER–mitochondrial Ca^2+^ transfer at the MAMs can be altered by different fine-tuning mechanisms. These mechanisms often involve oncogenes and tumor suppressors. An important tool to regulate the activity of the Ca^2+^-signaling proteins at the MAMs is phosphorylation. Akt/PKB-mediated phosphorylation is known to suppress Ca^2+^ release via the IP_3_R. However, this phosphorylation is counteracted by both PTEN, in a direct way, and PP2A, in an indirect way. This indirect mechanism consists of recruitment of PP2A to the IP_3_R via PML. In turn, PP2A deactivates Akt/PKB by dephosphorylation and this relieves IP_3_R inhibition. In addition, Ca^2+^-signaling proteins like IP_3_R, VDAC1, and SERCA interact with other proteins, which change their Ca^2+^-signaling properties. Bcl-2-protein family members are among these proteins altering Ca^2+^ signals at the MAMs. Bcl-2 binds to the IP_3_R, inhibiting pro-apoptotic Ca^2+^ signaling, while Bcl-Xl interacts with VDAC1, inhibiting Ca^2+^ uptake through the OMM. p53, a master regulator of cell fate, on the other hand, interacts with SERCA, changing its oxidation state, thereby enhancing reuptake of Ca^2+^ in the ER. A third category of proteins that modifies Ca^2+^ signaling at the MAMs are those proteins that change the properties of the MAMs, e.g. ER-to-mitochondria distance at the MAMs. Mfn-2 in the ER is able to interact with Mfn-1 or Mfn-2 at the OMM, thereby tethering both organelles. On the other hand, there is FATE1 which has the opposite effect, namely spacing the mitochondria and the ER. The distance between ER and mitochondria at the MAMs is determined by these tethers and spacers and this, in turn, regulates the efficiency of ER–mitochondrial Ca^2+^ transfer

Given its critical role in cell fate and survival, the composition and functional properties of the MAMs have to be carefully regulated and controlled. This occurs through a distinct set of proteins with a variety of cell biological properties and functions. Some of these proteins are directly involved in Ca^2+^ signaling at the MAMs, e.g. the IP_3_R and VDACs^49,54,55^, whereas others alter ER–mitochondrial Ca^2+^ signaling by acting through modulation of these Ca^2+^-transport systems, e.g. promyelocytic leukemia protein (PML)^56^ or phosphatase and tensin homolog (PTEN)^57^. Other proteins modify the characteristics of the MAMs (e.g. their distance to the mitochondria), like the ER–mitochondria tethers mitofusin-2 (Mfn-2)^58,59^, the protein kinase RNA-like endoplasmic reticulum kinase (PERK)^60^, and the spacer protein fetal and adult testis expressed 1 (FATE1)^61^. We will focus on the proteins involved in ER–mitochondrial Ca^2+^ transfer at the MAMs, yet for a more extensive list of proteins present at the MAMs, we refer to ref. ^13^. For further insights on the role of Ca^2+^-transport systems in cell death and survival, we refer to ref. 62. All the MAM components discussed in this section are schematically depicted in Fig. 2.

Since ER–mitochondrial Ca^2+^ transfer occurs at the MAMs, it is not surprising that several intracellular Ca^2+^-transport systems reside at the MAMs. Both the IP_3_R and VDAC1 can be found at the MAMs, where they are connected to each other via glucose-related protein of 75 kDa (GRP75)^55^. Interestingly, isoform-specific functions for these channels at the MAMs have been discovered. Overexpression of VDAC1, VDAC2, as well as VDAC3 increased mitochondrial Ca^2+^ uptake, yet pro-apoptotic Ca^2+^ signals were only enhanced by VDAC1 overexpression^63^. Moreover, VDAC1 is the only VDAC isoform that co-immunoprecipitated with the IP_3_R^63^. Similarly, IP_3_R3 seems to be the isoform that preferentially transmits pro-apoptotic Ca^2+^ signals to the mitochondria via the MAMs^64,65^. Not only Ca^2+^-release channels are located at the MAMs, the sarco-/endoplasmic reticulum Ca^2+^ ATPase (SERCA) pump localizes to the MAMs as well^66–68^. SERCA pumps can influence the properties of the MAMs since their activity determines how fast Ca^2+^ is cleared from the micro-domain^69^. Moreover, SERCA pumps critically affect apoptotic sensitivity by controlling the steady-state filling level of the ER Ca^2+^ stores, which is determined by the balance between ER Ca^2+^ leak and ER Ca^2+^ uptake^66,69–72^. Also for SERCA, isoform-specific functions exist: ER stress causes induction of SERCA1T, a truncated splice-variant of SERCA1. SERCA1T is a determinant of ER leakiness, thereby promoting Ca^2+^ transfer to the mitochondria and thus supporting pro-apoptotic Ca^2+^ signaling^73,74^.

As will be discussed further on, expression levels of the Ca^2+^-signaling proteins critically determine the cell’s Ca^2+^-signaling properties. Furthermore, ER–mitochondrial Ca^2+^ signaling is fine-tuned by various oncogenes and tumor suppressors^75,76^. These proteins may induce post-translational modifications that alter the Ca^2+^-signaling properties of proteins at the MAMs^77^. Particularly, phosphorylation of the IP_3_R is a critical factor: Akt/protein kinase B (PKB) suppresses IP_3_R-mediated Ca^2+^ release through phosphorylation^78^, while tumor suppressors PTEN (direct dephosphorylation of IP_3_R)^57^ and PML (indirect dephosphorylation via sequestration of protein phosphatase 2A (PP2A) and subsequent Akt/PKB inhibition)^56^ counteract this. Also, SERCA is a target for post-translational modification: p53 changes SERCA’s oxidative state, promoting its ER Ca^2+^-uptake activity and thus altering the net flux of Ca^2+^ released from the ER^66,79,80^.

In addition, complex formation between Ca^2+^-transport proteins like IP_3_Rs and VDAC1 at the MAMs and tumor suppressors or oncogenes influences ER–mitochondrial Ca^2+^ transfer. Notably, several Bcl-2-protein family members, critical regulators of apoptosis, were shown to be present at the MAMs^81,82^ and can modify IP_3_R-mediated Ca^2+^-release^77,83,84^. Bcl-2, an anti-apoptotic protein of the Bcl-2-protein family, inhibits the IP_3_R directly by binding with its Bcl-2 homology (BH) 4 domain to the central, modulatory domain of the IP_3_R^85–87^. The BH4 domain also participates in overall stability of the Bcl-2 protein, affecting its IP_3_R-inhibitory properties^88^. Bcl-2’s transmembrane domain is necessary for efficient in cellulo IP_3_R inhibition^89^. Indirectly, Bcl-2 changes IP_3_R activity by providing a docking place at the IP_3_R for protein phosphatase 1 (PP1), which inhibits IP_3_R function by dephosphorylating the receptor^90^. Bcl-2 also regulates IP_3_R function by docking dopamine- and cAMP-regulated phosphoprotein of 32 kDa, a PP1 inhibitor, and the protein phosphatase calcineurin in a complex on the IP_3_R^91^. Thus, IP_3_R activity is regulated by a negative feedback mechanism that prevents excessive, pro-apoptotic Ca^2+^ release from the ER. Besides Bcl-2, also Bcl-Xl and Mcl-1 can modulate IP_3_R activity^92–94^. An extended discussion on the modulation of Ca^2+^ signaling by Bcl-2-protein family members can be found elsewhere^82,95^.

Besides the IP_3_Rs, Bcl-2-family members can also target mitochondrial Ca^2+^-transport systems, including VDAC1. VDAC1/Bcl-2 complex formation inhibits VDAC1’s function in mitochondrial Ca^2+^ transport^96,97^. The BH4 domain of Bcl-2 appeared to play a critical role in VDAC1 regulation^98^. Follow-up work revealed that the BH4 domain of Bcl-Xl is more effective in targeting and modulating VDAC1 than the BH4 domain of Bcl-2^81^. Consequently, while both the BH4 domains of Bcl-2 and Bcl-Xl could prevent mitochondrial Ca^2+^ uptake, BH4-Bcl-2 acted at the level of IP_3_Rs, while BH4-Bcl-Xl acted at the level of VDAC1^81^. To summarize, ER–mitochondrial Ca^2+^ transfer at the MAMs can be influenced through IP_3_R-mediated Ca^2+^ release, VDAC1-mediated mitochondrial Ca^2+^ uptake, or modulation of the SERCA activity.

In addition to this, ER–mitochondrial Ca^2+^ transfer is altered by the number of MAMs and the distance between the ER and the OMM at the MAMs^99,100^. Proteins that hold both membranes together are typically called tethers. An example of a tether at the MAMs is the GTPase Mfn-2 involved in mitochondrial fusion. Mfn-2 tethers ER and mitochondria through homo- and heterotypic interactions with Mfn-2 and Mfn-1, respectively^59,101^. The importance of tethering was shown in Mfn-2-knockout cells, where ER–mitochondrial Ca^2+^ transfer was severely reduced^101^. Yet, the role of Mfn-2 as a tether has been debated, since another study showed that ablation of Mfn-2 does not impair the ER–mitochondrial connection but contrarily, tightens it^58,101^. Here, Mfn-2 was proposed as a spacer that increases the distance at the MAMs and reduces ER–mitochondrial signaling. A typical spacer is FATE1, a cancer testis antigen, which is normally only expressed in the testis^61^. However, in certain cancers, FATE1 becomes upregulated and causes MAMs alterations^61^.

## Rewiring Ca^2+^ signaling in cancer cells

The different possible layers of regulation of Ca^2+^ signaling impart an enormous flexibility to the cell concerning fine-tuning cellular processes in response to internal and external stimuli. However, this sensitive system can be hijacked to drive malignant transformations in the cell^6^. It is known that several types of cancer cells undergo an extensive rewiring of their Ca^2+^-signaling machinery, favoring oncogenesis^6,102,103^. At the level of the ER and the mitochondria, expression levels of Ca^2+^-signaling proteins, including VDAC1, IP_3_R, and SERCA, are often altered in cancer cells. For instance, VDAC1 expression levels are correlated with tumor growth and invasion in several types of cancer, e.g. non-small cell lung cancer and cervical cancer^104,105^. In this regard, recently, genetic disruption of VDAC1 in cells from cancer xenograft models displayed decreased mitochondrial membrane potential and ATP content with a consequent low migration rate and tumor regression^106,107^. Another example includes IP_3_R1 downregulation in bladder cancer cells, which attenuates cisplatin-mediated apoptosis through a decrease in ER–mitochondrial Ca^2+^ uptake, preventing mitochondrial Ca^2+^ overload^108,109^. Remodeling of Ca^2+^ signaling in tumorigenesis is also documented by the significant reduction or loss of the SERCA3 isoform in transformed colonic epithelial cells^110^. Different mechanisms may be responsible for the change in expression levels. Recently, two novel mechanisms dysregulated in several cancer types have been discovered to impact the proteasomal turnover and thus stead-state expression levels of IP_3_R3 and the apoptotic sensitivity of cells^111^. (i) The tumor suppressor PTEN competes with F-box/LRR-repeat protein 2 (FBXL-2), an E3 ubiquitin ligase component belonging to the Skip-Cullin1-F-box protein family^112^, for binding to IP_3_R3, thereby slowing down FBXL-2-mediated proteasomal degradation of IP_3_R3^113^. This represents a novel mechanism by which loss of PTEN allows cancer cells to evade apoptosis, since pro-apoptotic mitochondrial Ca^2+^ transfer becomes impaired due to downregulation of the IP_3_R3. (ii) The tumor suppressor BRCA1-associated protein 1 (BAP1), a deubiquitylating enzyme, promotes ER–mitochondrial Ca^2+^ transfer by stabilizing the IP_3_R3^114^. BAP1 function is particularly impaired during prolonged environmental stress, associated with acquired inactivating mutations in BAP1 genes. Loss of BAP1 results in IP_3_R3 downregulation, hampering the effective apoptotic clearance of damaged cells and favoring oncogenesis and malignant cell survival.

While MCU is not residing at the MAMs, its expression can be controlled in a tumor-specific manner, e.g. via microRNAs (miR)^115^. As such, miR-25, targeting MCU, was overexpressed in colon cancer cell lines and tumor samples, decreasing MCU expression compared to non-tumorigenic cells. Moreover, miR-25 overexpression in HeLa cells reduced mitochondrial Ca^2+^ accumulation, resulting in apoptosis resistance. In contrast, antagonizing miR-25 expression using antagomirs re-sensitized colon cancer cells to Ca^2+^-dependent apoptotic stimuli, like H_2_O_2_ and ceramide. Interestingly, MCU may prevent tumor cell survival in an early stage, but can become a pro-malignant factor in late-stage tumors, like triple negative breast cancer cells^116^. These cells express high levels of MCU, correlating with tumor size and lymph node infiltration, which negatively impact survival outcome. The mechanisms involved MCU-dependent uptake of Ca^2+^ into the mitochondrial matrix and subsequent generation of ROS that stabilized hypoxia-inducible factor-1α, a transcription factor driving the expression of genes involved in cancer migration and invasion^116,117^.

Furthermore, oncogenes like Akt/PKB and FATE1, and tumor suppressors like PML and PTEN, can play additional roles in the development of cancer via Ca^2+^-signaling modulation^108^. A striking example of this is apoptotic resistance. Since mitochondrial Ca^2+^ overload is involved in apoptotic cell death, modifying ER–mitochondrial Ca^2+^ transfer at the MAMs alters apoptotic sensitivity^48^. Cancer cells can gain resistance against cell death, e.g. by overexpressing proteins that suppress IP_3_R-mediated Ca^2+^ signaling, like Akt^78^, or by increasing the intermembrane distance at the MAMs (e.g. FATE1), thereby rendering ER–mitochondrial Ca^2+^ transfer less efficient^61^. These mechanisms are not only supporting basic cancer cell characteristics, but also underlie resistance against chemotherapy. Cell death induction strategies still play a central role in the fight against cancer. While selective oncogene inhibitors, like venetoclax/ABT-199 for Bcl-2 inhibition, emerged and entered the clinic^118^, chemotherapy remains a very effective way to eradicate tumor cells by triggering cell death^119^.

Besides ER–mitochondrial Ca^2+^ signaling, Ca^2+^ fluxes across the plasma membrane can affect cancer properties as well. We would like to refer to other excellent reviews regarding this topic^120–122^, as in this section, we would like to focus on chemotherapeutic drugs that act on Ca^2+^ signaling at the MAMs.

## Chemotherapy and Ca^2+^ signaling in cancer cells

Ca^2+^ signaling appears to be a major contributor to the cytotoxic effects of chemotherapy. Many chemotherapeutic agents trigger a rapid onset of cytosolic [Ca^2+^] rises^123^. Furthermore, shifts in cytosolic [Ca^2+^] have been proposed as early markers for cytotoxicity in cells in response not only to H_2_O_2_ or staurosporine but also to chemotherapeutics like gossypol or arsenic trioxide (ATO)^123^. The mechanism of these early cytosolic [Ca^2+^] elevations is not always fully understood, but may in part depend on the presence of p53. Upon chemotherapeutic treatment, extra-nuclear p53 can accumulate at the ER membranes where it binds SERCA and activates its ER Ca^2+^-uptake activity^66,79, 80,124,125^. Thus, SERCA activation will augment the ER Ca^2+^-store content, overfilling the ER with Ca^2+^ and thus increasing the likelihood for spontaneous ER Ca^2+^-release events^126^. However, the occurrence of shifts in cytosolic [Ca^2+^] may not be a general phenomenon, as on-target Bcl-2 inhibitors, like ABT-737^127^ and venetoclax/ABT-199^128^, do not trigger these early cytosolic [Ca^2+^] elevations, even not in cancer cells that are dependent on Bcl-2 for their survival. The reason for these varying responses are not fully understood, but may actually relate to the mechanism of action of the drug applied and in particular whether p53 is involved.

**Fig. 3:**
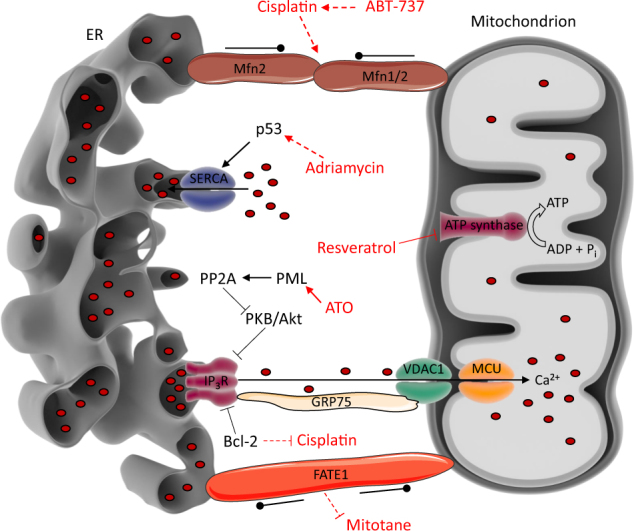
The action of chemotherapeutic agents at the MAMs. Arrow-headed full lines indicate a stimulatory effect, while bar-headed full lines indicate an inhibitory effect. Dashed lines indicate an indirect effect. Dot-headed lines indicate a tethering or a spacing effect, depending on the inward- or outward-facing dots, respectively. This figure constitutes an overview of the chemotherapeutics discussed in this review and how they act on the MAMs. Resveratrol is an inhibitor of the ATP synthase. This inhibitory effect leads to less ATP being available for SERCA pumps to ensure rapid reuptake of Ca^2+^ in the ER, creating a high local Ca^2+^ concentration. This, together with an increased ER–mitochondrial tethering, underlies the cancer cell-specific killing by resveratrol. ATO, another chemotherapeutic agent, increases PML levels at the MAMs. This restores ER–mitochondrial Ca^2+^ transfer in cancer cells that have a decreased or impaired PML activity and consequently suppresses pro-survival autophagic flux. A third chemotherapeutic drug, cisplatin, covalently binds to DNA, inducing DNA damage and causing apoptotic cell death, involving ER–mitochondrial Ca^2+^ signaling. However, cancer cells overexpressing Bcl-2 are more resistant to cisplatin-induced cell death, seemingly via a dual mechanism: the inhibition of Ca^2+^ release from the ER and the inhibition of an increase in ER–mitochondrial contact points resulting from cisplatin treatment. In this sense, administering ABT-737, a Bcl-2-inhibiting BH3 mimetic, to cancer cells, restored sensitivity to cisplatin. Furthermore, there is adriamycin, which renders ER–mitochondrial Ca^2+^ transfer more efficient by enriching p53 at the SERCA pumps. This leads to an increased activity of SERCA, increasing the ER Ca^2+^ levels and sensitizing cells towards apoptosis. Lastly, mitotane is an inhibitor of SOAT1, resulting in increased free cholesterol and lipid-induced ER stress. However, sensitivity towards mitotane is dependent on the expression levels of FATE1, a spacer protein at the MAMs. Increased levels of FATE1 are responsible for decreased mitochondrial Ca^2+^ uptake and in this way render cancer cells less sensitive towards apoptosis

Here, we will discuss the chemotherapeutic agents that act via ER–mitochondrial Ca^2+^ signaling. These chemotherapeutics are summarized in Table 1, whereas a schematic representation of their function at the MAMs is provided in Fig. 3.

**Table 1:** Overview of the different chemotherapeutic agents that act through mechanisms related to the MAMs and/or targets localized at the MAMs

Chemotherapy	Target protein	Mechanism of action at MAMs	Functional effect	Cancer type	Reference
Arsenic trioxide	PML	Elevating PML levels, which results in an increased IP_3_R-mediated ER–mitochondrial Ca^2+^ transfer	Repression of autophagy	Acute promyelocytic leukemia	^17^
Cisplatin	Unknown	Increasing ER–mitochondria contact sites and subsequent mitochondrial Ca^2+^ overload	Apoptosis	Ovarian cancer, non-small cell lung cancer & bladder cancer	^140^
ABT-737	Bcl-2 & Bcl-Xl	Alleviating the decrease in IP_3_R-mediated Ca^2+^ release by Bcl-2	(Re)sensitization to cisplatin therapy	Ovarian cancer & cholangiocarcinoma	^143,158^
		Antagonizing the inhibitory action of Bcl-2 on MAM formation induced by cisplatin			
Resveratrol	ATP synthase	Augmenting mitochondrial Ca^2+^ due to impaired SERCA activity in the MAMs as a consequence of ATP synthase inhibition	Apoptosis	Broad spectrum	^166^
Adriamycin	p53	Increasing SERCA activity and ER Ca^2+^ loading via p53, which is enriched at ER & MAMs	Apoptosis	Broad spectrum	^66^
Mitotane	SOAT1	Provoking the accumulation of toxic cholesterol lipids	Apoptosis	Adrenocortical carcinoma	^61^

### Arsenic trioxide

Acute promyelocytic leukemia (APL) is almost always characterized by a t(15;17) chromosomal translocation, resulting in a PML/retinoic acid receptor (RAR) α fusion protein that hinders the differentiation of hematopoietic cells by inhibiting gene transcription^129,130^. APL patients are mostly treated by a combination of all-*trans* retinoic acid and ATO therapy, which stimulates APL cell differentiation by triggering proteasomal degradation of the PML/RARα fusion protein^129,131–133^. This treatment approach results in high APL cure rates, reflected by high complete remission and overall survival percentages^129,132^.

ATO also influences ER–mitochondrial Ca^2+^ signaling, thereby repressing autophagy in cancer cells^17^. Autophagy is an important pro-survival pathway in malignant cells that experience oncogenic stress, as missing nutrients can be delivered to the cells via this process^17,134^. The tumor suppressor PML, which is localized at the MAMs, represses autophagy by promoting ER–mitochondrial Ca^2+^ transfer and mitochondrial respiration^17^. Hence, PML is often downregulated in cancer cells^135^. Loss of PML results in reduced IP_3_R-mediated Ca^2+^ transfer from the ER to the mitochondria, leading to decreased mitochondrial Ca^2+^ levels, thereby diminishing mitochondrial respiration and ATP production^17^. This triggers activation of AMP-activated protein kinase, which stimulates pro-survival autophagy by a mechanism involving mechanistic target of rapamycin and unc-51-like kinase 1 pathways^136,137^. Interestingly, this autophagic process is repressed in APL cells treated with ATO^17^. Short-term treatment of these cells with ATO promotes the selective degradation of the PML/RARα fusion protein, but not of PML. Furthermore, exposure to ATO increases the PML levels at ER–mitochondria contact sites in a p53-dependent manner (Fig. 3). Besides a block in autophagy, ATO-treated cells also displayed reduced resistance to metabolic stress^17^. Furthermore, this study implicates that the response of tumor cells characterized by loss of PML to chemotherapeutic agents can be improved by inhibiting autophagy.

### Cisplatin

Cisplatin is a chemotherapeutic agent used to treat numerous human cancers, including lung, ovarian, head and neck, bladder, and testicular cancer^138^. The anticancer activity of this platinum-based drug has been linked to its ability to covalently bind purine residues on DNA^138,139^. This interaction causes DNA damage, interferes with DNA repair mechanisms, and blocks cell division, ultimately leading to apoptotic cell death. Unfortunately, cisplatin treatment has been associated with considerable side effects, like cardiotoxicity, hepatotoxicity, nephrotoxicity, and toxicity of other organs, and drug resistance is often acquired during therapy as well^138^. To overcome these obstacles, combination therapies of cisplatin with other anti-cancer drugs, including paclitaxel, doxorubicin, and gemcitabine, form the basis for treatment of many human cancers^138^.

Interestingly, the ER–mitochondrial Ca^2+^ signaling pathway contributes to cisplatin-induced cell death. Treatment of SKOV3 human ovarian cancer cells with cisplatin increased the number of ER–mitochondria contact sites, causing a Ca^2+^ flow from the ER to the mitochondria (Fig. 3)^140^. This resulted in high mitochondrial Ca^2+^ levels, which triggered apoptosis in the cisplatin-treated ovarian cancer cells. Furthermore, the expression level of the anti-apoptotic protein Bcl-2, which is overexpressed in many tumors and drives tumorigenesis and chemoresistance, seems to be a determinant for the sensitivity of cancer cells to cisplatin (Fig. 3). In non-small cell lung cancer and bladder cancer, cisplatin sensitivity could be enhanced by downregulating Bcl-2^141,142^. In addition, downregulation of Bcl-2 in SKOV3 cells with siRNA increased the cytoplasmic and mitochondrial Ca^2+^ levels as well as the number of ER–mitochondria contact points after cisplatin treatment, thereby increasing the sensitivity to the chemotherapeutic agent^143^. Hence, Bcl-2 seems to form a potential therapeutic target to improve cisplatin therapy of ovarian cancer cells. Additionally, in neuroblastoma cells, cisplatin-induced cell death was preceded by a rise in cytosolic [Ca^2+^]^144^. Cisplatin treatment also increased the expression levels of several Ca^2+^-transport systems, including IP_3_R3, RyR3, and the S100 Ca^2+^-binding protein A6. Therefore, cisplatin-induced cell death can be enhanced by pharmacological modulators of Ca^2+^-regulatory proteins, like the SERCA inhibitor thapsigargin^144^.

### BH3 mimetics

BH3 mimetics are a class of anti-cancer drugs inhibiting the function of anti-apoptotic Bcl-2-protein family members like Bcl-2, Bcl-Xl, and Mcl-1^145–147,148^. This causes pro-apoptotic Bcl-2-family members, which are sequestered by their anti-apoptotic counterparts through a hydrophobic cleft, consisting of the BH1, BH2, and BH3 domain, to be released, resulting in apoptosis^145,146^. This is an effective anti-cancer therapy in cancers that rely on an upregulation of the anti-apoptotic Bcl-2-family proteins for their survival. Several molecules have been developed as BH3 mimetic drug, notably ABT-737, which targets the hydrophobic cleft of both Bcl-2 and Bcl-Xl, its orally available analog ABT-236 (navitoclax) and ABT-199 (venetoclax), which solely targets the hydrophobic cleft of Bcl-2^145^. While these drugs are specifically developed to suppress the canonical, anti-apoptotic function of the Bcl-2-protein family members at the mitochondria, it seems that their intracellular effects are more complex.

Thrombocytopenia is an important side effect caused by ABT-737 and ABT-263, since these BH3 mimetic drugs inhibit the function of Bcl-Xl, which is essential for platelet formation and survival^149–151^. Dysregulation of intracellular Ca^2+^ homeostasis might underlie ABT-737- and ABT-263-induced thrombocytopenia, as addition of ABT-263 to platelets triggered an acute rise in cytosolic Ca^2+^ levels^149^. However, a direct link between deranged Ca^2+^ signaling and platelet dysfunction was not provided since this effect was only observed upon addition of relatively high ABT-263 concentrations (10 µM), whereas platelet function was already decreased at much lower concentrations (100 nM–1 µM)^149^. Furthermore, prolonged treatment of platelets with ABT-263 and ABT-737 depleted the intracellular Ca^2+^-storage organelles^149,152^. However, it was not clear whether the effects on Ca^2+^ signaling were the cause of platelet apoptosis or whether they were the consequence of platelets being in late-stage apoptosis due to Bcl-Xl inhibition. Our lab excluded a direct impact of ABT-737 on intracellular Ca^2+^ signaling, since ABT-737 application did not affect thrombin-induced Ca^2+^ signaling in platelets nor ATP-induced Ca^2+^ signaling in HeLa cells^127^. Moreover, SERCA activity and IP_3_R-mediated Ca^2+^ release were unaffected by ABT-737. These results argue against a proximal role of Ca^2+^-signaling dysregulation in platelet dysfunction and apoptosis induced by ABT-737.

On the other hand, for ABT-199/venetoclax, which only targets the hydrophobic cleft of Bcl-2, no evidence was found for a perturbation of Ca^2+^ homeostasis^128,148,153^. In several human and mouse cell models, ABT-199 did not trigger cytosolic Ca^2+^ release events by itself nor did it affect agonist-induced IP_3_R-mediated Ca^2+^ signaling^128^. Also, clearance of Ca^2+^ from the cytosol after agonist application was not affected. Furthermore, ABT-199 did not interfere with the inhibition of IP_3_R caused by overexpression of Bcl-2^128^. Nevertheless, it seems that there is an interplay between ABT-199-induced cell death in Bcl-2-dependent cancer cells and basal Ca^2+^ signaling, since chelating intracellular Ca^2+^ using BAPTA-AM enhanced ABT-199-induced cell death^128^. The mechanisms underlying this phenomenon remain unclear, but might be due to downregulation of anti-apoptotic Bcl-2-family members or upregulation of pro-apoptotic Bcl-2-family members. Furthermore, Bcl-2-dependent cancer cells can be sensitized towards ABT-199 by the application of BIRD-2^154^, a BH4-domain inhibitor of Bcl-2 that triggers toxic Ca^2+^-release events and apoptosis in various cancer cells, including chronic lymphatic leukemia, diffuse large B-cell lymphoma, multiple myeloma, follicular lymphoma, and lung cancer cells^86,154–156^. Further, it appears that BIRD-2 upregulates Bim in a Ca^2+^-dependent manner, thereby likely accounting for an increased sensitivity of the cells towards BH3 mimetics like ABT-199 application^154,157^. As such, BIRD-2 and ABT-199 can act synergistically to trigger cell death in Bcl-2-dependent cancers. Finally, it appears that cancer cells that are less sensitive to BH3 mimetics are more sensitive to BIRD-2 and vice versa^154,156,157^.

Interestingly, ABT-737 can enhance the chemotherapeutic effectivity of cisplatin in cholangiocarcinoma (CC) as well as in ovarian cancer cells^143,158^. In the latter, ABT-737 treatment increased the cisplatin-induced growth inhibition and apoptosis in cisplatin-resistant cells, hence restoring their sensitivity to the chemotherapeutic agent^143^. Combination therapy with cisplatin and ABT-737 of the cisplatin-resistant ovarian cancer cells increased the number of ER–mitochondria contact sites induced by cisplatin, stimulating Ca^2+^ transfer from the ER to the cytosol and the mitochondria (Fig. 3). Hence, ABT-737 can reverse cisplatin resistance of ovarian cancer cells by enhancing ER-associated and mitochondria-mediated apoptosis^143^. Furthermore, ABT-737 sensitized CC cells to cisplatin therapy by regulating mitochondrial dynamics^158^. ABT-737 stimulated CC cells to undergo apoptosis after cisplatin treatment by promoting mitochondrial fission and inducing mitophagy. Therefore, ABT-737 combined with cisplatin might be an effective strategy to treat CC patients^158^. This also provokes the question in what manner Bcl-2-protein family members regulate the dynamics of the MAMs in cancer.

Of note, earlier versions of Bcl-2-inhibiting molecules can dysregulate intracellular Ca^2+^ homeostasis. HA14-1 and stabilized HA14-1s can deplete ER Ca^2+^ stores by inhibiting the SERCA Ca^2+^ pump^127,159^. In a separate study, both HA14-1 and BH3I-2′ trigger Ca^2+^ release from the ER of pancreatic acinar cells through IP_3_R- and RyR-mediated mechanisms, elevating cytosolic Ca^2+^ levels, a feature that contributes to their cell death properties^160^. Here, it was proposed that dissociation of Bax from Bcl-2 using these drugs sensitize IP_3_Rs and RyRs to cytosolic Ca^2+^. Excitingly, the ability of HA14-1 and BH3I-2′ to increase cytosolic Ca^2+^ levels in pancreatic acinar cells was strictly dependent on the presence of Bax, while the presence of Bcl-2 and Bak was not critical for this process. It was proposed that Bax, released from Bcl-2, can induce Ca^2+^ leak from the ER, either by itself or by acting on ER Ca^2+^-leak channels like IP_3_Rs^161^. Yet, further work using selective Bcl-2 inhibitors is needed to validate this model. A detailed discussion on the role of BH3 mimetics and Ca^2+^ signaling is provided elsewhere^148,162^.

### Resveratrol

Resveratrol is a natural polyphenol produced in response to stressful conditions by various plant species. It is found in several foodstuffs, including grapes, mulberries, and peanuts, and has been attributed beneficial health effects since the early 1990s^163–165^. It is known as a multi-target agent exhibiting antioxidant, anti-inflammatory, and immunomodulatory activities. This pleiotropic compound affects cell proliferation, differentiation, apoptosis, and autophagy and attenuates many age-related chronic complications, such as metabolic, cardiovascular, and neurodegenerative diseases^164,165^. Furthermore, resveratrol has been used as a chemopreventive and chemotherapeutic agent in many types of cancer^163–165^. For instance, it has been used in clinical trials conducted in patients with colon, colorectal, and gastrointestinal cancers as well as in a trial examining the effects of resveratrol in the prevention of cancer in healthy participants^163^. Remarkably, resveratrol functions as a specific anticancer agent with limited toxicity in normal cells^163^. However, the exact mechanism by which resveratrol specifically kills cancer cells remains unclear.

Recently, it was suggested that the difference in ATP demand between cancer and somatic cells underlies the cancer cell-specific toxicity of resveratrol^166^. Cancer cells are characterized by a very high ATP demand at the ER because of immense protein folding activities going on at this organelle^167,168^. Therefore, tethering of the ER and the mitochondria, which produce ATP via the ATP synthase localized in the IMM, is strongly enriched in cancer cells, warranting high ATP levels in the proximity of the ER^169,170^. However, resveratrol, which acts as an inhibitor of the F1 subunit of the ATP synthase^171–174^ exploits the enhanced ER–mitochondria coupling in malign cells to kill these cells exclusively (Fig. 3). As a result of ATP synthase inhibition in resveratrol-treated cells, ATP formation is reduced, by which the high energy demand of cancer cells is not met anymore^166^. As a consequence of the reduced ATP content at the mitochondria, SERCA activity within the MAMs is decreased, hampering Ca^2+^ reuptake into the ER, provoking not only an accumulation of Ca^2+^ in the micro-domain between ER and mitochondria^166^, but also leading to a depletion of the ER via the Ca^2+^-leak channels^175^. Both phenomena result in a high local Ca^2+^ concentration and because of the enforced ER–mitochondria coupling in cancer cells, mitochondrial Ca^2+^ accumulation is consequently enhanced upon treatment with resveratrol. Due to the fact that cancer cells are more sensitive to ATP synthase inhibition than healthy cells, resveratrol will especially trigger apoptotic cell death via mitochondrial Ca^2+^ overload in those cells^166^.

### Adriamycin

Adriamycin, also known as doxorubicin, is an anthracycline-type drug that has been used in cancer therapy for many years^176,177^. It is characterized by a broad-spectrum antineoplastic activity, although its mechanism of action is complex. Adriamycin inhibits topoisomerase II, intercalates in DNA, and generates free radicals, hence inhibiting biosynthesis of macromolecules and leading to oxidative stress^177–179^. Ultimately, adriamycin treatment results in apoptotic cell death. This chemotherapeutic agent is commonly used to treat various types of cancer, including breast, ovarian, bladder, stomach, and lung cancer^177^. However, the applicability of adriamycin as anticancer therapy is restricted due to its severe toxic effects in healthy tissues^176,177,179^. Especially cardiotoxicity forms a major concern during adriamycin therapy, limiting the dose that can be administered to patients^177,180^. To circumvent the adverse effects of adriamycin, several drug delivery systems have been used^176,177,179^. For instance, adriamycin-induced toxicity is decreased when using liposomal, nanoparticle, or hydrogel drug formulations.

Adriamycin also renders cells more prone to programmed cell death by influencing the ER–mitochondrial Ca^2+^ signaling axis^66,79,181^. The tumor suppressor p53, an important transcription factor regulating DNA repair, cell-cycle arrest, and apoptosis, modulates Ca^2+^ homeostasis by stimulating the SERCA pump located at the ER and the MAMs^66^. As a consequence, Ca^2+^ accumulation in the ER is enhanced. Interestingly, treatment of cancer cells with adriamycin caused an enrichment of p53 at the ER and the MAMs (Fig. 3)^66,79^. This induction of p53 by adriamycin led to higher ER Ca^2+^ levels and higher cytosolic and mitochondrial [Ca^2+^] increases evoked by agonist stimulation^66^. Moreover, Ca^2+^ transport from the ER to the mitochondria was increased by adriamycin, allowing apoptotic stimuli to rapidly overload the mitochondria with Ca^2+^, resulting in apoptotic cell death. Hence, chemotherapeutic agents like adriamycin boost toxic ER–mitochondrial Ca^2+^ signaling through modulation of the Ca^2+^ homeostasis at the MAMs, thereby triggering apoptotic cell death in cancer cells^66,79^.

Another way in which Ca^2+^ signaling is able to contribute to the sensitivity of cancer cells to chemotherapeutics, is the induction of autophagy^182^. Recently, valproic acid was found to reduce the intracellular availability of IP_3_, hence blocking Ca^2+^ transfer to the mitochondria and altering the AMP-activated protein kinase 1/2–mechanistic target of rapamycin pathway. This lack of ER–mitochondrial signaling induced autophagy, thereby sensitizing cancer cells to adriamycin^182^.

### Lipid-interfering strategies

A recent insight in tumor biology is the occurrence of dysregulation of lipid metabolism in cancer cells and its importance to several aspects of cancer cell function and survival. Consequently, disrupting or altering lipid homeostasis in cancer cells might be an efficient way of inducing cell death^183^. One of the ways to induce apoptosis in this manner in cancer cells is the use of chemotherapeutics that increase intracellular, free cholesterol^183^. Alkyl phospholipids were observed to hinder cholesterol transport from the mitochondria to the ER, avoiding its esterification, whereas cholesterol synthesis and incorporation was increased at the same time in glioblastoma cells^184^. In macrophages it was found that this free cholesterol accumulates at the ER, inhibiting SERCA pumps and causing depletion of the ER. This results in an increased transfer of Ca^2+^ to the mitochondria and subsequent apoptosis^185^.

Another chemotherapeutic drug that acts on lipid metabolism is mitotane, a derivative of the insecticide dichlorodiphenyl-trichloroethane. Mitotane is the only chemotherapeutic drug approved for the treatment of adrenocortical carcinoma (ACC), one of the deadliest endocrine malignancies^186–189^. In ACC cells, but not in non-adrenal cancer tissues, mitotane counteracts tumor growth and steroid hormone production^186,190^. These effects of mitotane are believed to be the result of the inhibition of the sterol-*O*-acyl-transferase 1 (SOAT1), an enzyme that protects cells against the harmful effects of free cholesterol by transforming it into cholesterol esters. Because of SOAT1 inhibition, mitotane therapy leads to the accumulation of toxic lipids, including free cholesterol and oxysterols, inside ACC cells, which triggers lipid-induced ER stress^190^. Moreover, in ACC tissue samples, SOAT1 expression correlated with the response to mitotane treatment.

Interestingly, mitotane-induced apoptosis in ACC cells also depends on FATE1 expression^61^. Under physiological conditions, FATE1 expression is restricted to the testis and adrenal gland, while FATE1 overexpression is observed in a variety of cancers^191,192^. In ACC cells, FATE1 expression is controlled by the steroidogenic factor-1 (SF-1), a transcription factor that is important for adrenal development and plays a role in the formation of adrenocortical tumors^61,191^. FATE1, localized at the MAMs where it uncouples the ER and mitochondria, decreases mitochondrial Ca^2+^ uptake in ACC cells^61^. In this way, FATE1 protects cancer cells from Ca^2+^-dependent apoptotic stimuli. Furthermore, FATE1 expression conferred mitotane resistance to ACC cells, when this chemotherapeutic drug was used in a dose that falls inside the therapeutic window for ACC patients, whereas knockdown of FATE1 in these cells increased the sensitivity to the drug^61^. FATE1 probably protects against mitotane-induced apoptosis because of its localization in the MAMs^61^. Mitotane inhibits the SOAT1 enzyme^190^, which is also localized in the MAMs, resulting in the accumulation of toxic cholesterol lipids. Hence, in the presence of FATE1 mitotane-mediated SOAT1 inhibition may be less efficient (Fig. 3)^61^. Interestingly, FATE1 expression in ACC tumor cells can even be used as a prognosis indicator since FATE1 expression is inversely correlated with the overall survival of ACC patients^61^.

## Conclusion

In conclusion, MAMs form important intracellular signaling platforms, allowing for Ca^2+^-encoded messages between the ER and the mitochondria. This ER–mitochondrial Ca^2+^ exchange can be altered during cancer development to promote cancer hallmarks like evasion of apoptosis, excessive cell proliferation, and a metabolic rewiring. In addition, many chemotherapeutics act via Ca^2+^ signaling at the MAMs (Fig. 3). Moreover, chemotherapeutics can interfere with the function of oncogenes and tumor suppressors, thereby altering ER–mitochondrial Ca^2+^ transfer. In this sense, chemotherapeutics that modify ER–mitochondrial Ca^2+^ signaling can be used to increase the response of cancer cells towards therapeutics that harbor a Ca^2+^ component in their working mechanism.
